# Comparison of the Factors Influencing Pulmonary Arterial Pressure in Smoker and Non-smoker COPD Patients with Pulmonary Hypertension

**Published:** 2019-01

**Authors:** Abolhasan Halvani, Hamidreza Haddad

**Affiliations:** 1 Department of Internal Medicine, School of Medicine, Yazd Medical Science Branch, Islamic Azad University, Yazd, Iran.; 2 Department of Internal Medicine, Qazvin University of Medical Sciences, Qazvin, Iran.

**Keywords:** Pulmonary hypertension, Smoker, COPD, Non-smoker

## Abstract

**Background:**

There are several prognostic factors in patients with Chronic Obstructive Pulmonary Disease (COPD) that include Forced expiratory volume in one second (FEV1), Body Mass Index (BMI), dyspnea severity, exercise capacity and Pulmonary Hypertension (PH). PH is one of the most important factors. PH pathogenesis in patients with COPD has not been clarified thoroughly and factors such as alveolar hypoxemia, polycythemia, acidosis and pulmonary vessels obstruction have been suggested. The authors assessed some of these contributing factors in smoker and non-smoker patients with COPD.

**Materials and Methods:**

This comparative-descriptive study included COPD patients suspected to have cor pulmonale without exacerbation in the last four weeks. Echocardiographic evaluation of Pulmonary Arterial Pressure (PAP) was done and Pulmonary Hypertension (PH) was defined as systolic Pulmonary Arterial Pressure (PAP) greater than 40 mmHg. Complete Blood Count (CBC) and Arterial Blood Gas (ABG) were also studied in all patients.

**Results:**

Echocardiography was done for 142 patients who were suspected to have PH and 110 patients had measurable PAP. All of the patients were in stage II – IV of COPD according to GOLD criteria. 90 patients had PH of which 47 were smokers and 43 were non-smokers. In smoker patients, significant correlation between PAP and PaO_2_ was seen (r=−0.291, p-value=0.047). But in non-smoker patients, this correlation was absent. A significant correlation between PAP and FEV1 (r=−0.341, P value=0.025) was seen in non-smoker patients. This correlation was absent in smoker patients. There was no correlation between PAP and hemoglobin, hemoglobin and FEV, and also PaO_2_ and FEV1 in smoker and non-smoker COPD patients.

**Conclusion:**

In non-smoker patients with COPD, degree of pulmonary parenchymal lesions and bronchial obliteration plays a more important role than hypoxia in the pathogenesis of pulmonary hypertension.

## INTRODUCTION

Pulmonary Hypertension (PH) is present in most of the patients with severe Chronic Obstructive Pulmonary Disease (COPD). Prognosis in patients with COPD can be estimated by Forced Expiratory Volume in one second (FEV1), Body Mass Index (BMI), severity of dyspnea and exercise capacity, but recent studies have shown that PH has a great role in prognosis of the patients with COPD. The greater is Pulmonary Arterial Pressure (PAP), the more is the number of exacerbations and even the length of hospital stay in these patients (1–3). Some recent studies with large study samples have shown that PAP may predict mortality in patients with COPD and is independent of airflow limitation. Patients with COPD and mean PAP more than 30 mmHg have a high mortality rate and those with mean PAP of 50 mmHg have 100% mortality rate (4,5).

According to the definition, PH is present when mean PAP is greater than 25 mmHg at rest or 30 mmHg with exercise. Mean PAP should be measured by right heart catheterization. Systolic pulmonary artery pressure is determined by Doppler echocardiography and formerly was accepted as PH when it was greater than 40 mmHg which is equal to a tricuspid regurgitation velocity of 3.0 to 3.5 m/sec (6,7). In patients with COPD, an increased incidence of right ventricular involvement may correlate with increasing severity of lung dysfunction. Right ventricular hypertrophy is present in 40 percent of patients with an FEV1 <1.0 L and in 70 percent of those with an FEV1 <0.6 L (8). However, the presence of hypoxemia, hypercapnia, and polycythemia also independently predict the development of right ventricular hypertrophy in COPD, although not as strongly as abnormal pulmonary mechanics (6). Among these, alveolar hypoxemia is one of the main causes (9). Many of the patients with COPD and FEV1 less than 50% of predicted have alveolar hypoxemia, but in some patients, alveolar hypoxemia occurs in patients (especially non-smokers) with FEV1 more than 50% of predicted due to pulmonary vascular obliteration. Elevation of PAP is also observed during exercise, nocturnal desaturation and acute exacerbations in COPD patients (10).

Most of the patients with COPD have history of cigarette smoking or other inhalational exposure. However, some patients develop COPD without an obvious risk factor (11,12). Clinical manifestation and natural history of COPD is slightly related to its etiology. In the present study, some of the contributing factors to PH in patients with COPD who are smokers and non-smokers were assessed and compared.

## MATERIALS AND METHODS

### Patients

This comparative-descriptive study included all COPD patients suspected to have cor pulmonale according to clinical, radiologic and electrocardiographic evidence on referral to respiratory outpatient clinic of Shahid Sadoughi Hospital, Yazd, Iran. All of the patients were in stage II – IV of COPD according to GOLD criteria. These patients had a history of cough, sputum, persistent dyspnea, cigarette smoking or other COPD risk factors. They had FEV1/FVC<70% and FEV1<80% of predicted. Patients with asthma history and post bronchodilator response (more than 12% increase in FEV1 after inhaling salbutamol 400 μg) were excluded (13,14).

The selected patients had no history of exacerbation during previous four weeks. Patients with nocturnal hypoxemia according to night-time pulse oximetry, sleepiness ≥ 10 according to Epworth Sleep Scale, past history of left sided heart failure, pulmonary emboli, chest wall disorders and drug history of calcium channel blockers, diuretics, methyldopa, angiotensin converting enzyme inhibitors and alpha adrenergic blockers were excluded from the study.

Ethical Research Committee of Yazd University of Medical Sciences assessed the study protocol and did not find any discordance with ethical rules. Participated patients were informed and signed a consent form.

### Echocardiography

Echocardiography was done by a cardiologist and PAP was measured. The echocardiography system was a Vivid 3 (General Electric, US) with 2MHz probe. When systolic Tricuspid Regurgitation (TR) was present during color Doppler echocardiography, its velocity was measured. All patients were laid down on the bed in supine position. Transducer positions for obtaining maximal velocity were apical, lower left parasternal and subcostal. Tricuspid jet regurgitation was used for calculation of PAP. [Systolic PAP = (4×Tricuspid jet velocity squared) + Right arterial pressure]. Systolic pulmonary artery pressure more than 40 mmHg was considered as PH. Investigations included arterial blood gas and complete blood count.

FEV1, FVC, and FEV1/FVC were measured by Spirolab III (MIR, Roma, Italy) and GOLD criteria were used to classify the severity of airflow limitation.

### Statistical analysis

The data was analyzed by SPSS program version 15 (SPSS Inc., Chicago III). Percentiles and mean and standard deviations were analyzed for each variable. The normal distribution of variables was assessed by Kolmogrov-Smirnov test. Data in two study groups were compared by T-test. Pearson test analyzed correlations of PAP with age, smoking history and indices of pulmonary function. P-values < 0.05 were considered significant.

## RESULTS

Echocardiography was done for 142 patients who were suspected to have PH and 110 patients had measurable PAP. All of the patients were in stage II – IV of COPD according to GOLD criteria. Of the total, 90 patients (69 male, 21 female) had PH and 47 patients (45 male, 2 female) had history of smoking, while 43 patients (24 male, 19 female) had no history of smoking (Table 1). Demographic and cardiopulmonary characters of COPD patients with PH are shown in table 2.

**Table 1.** Frequency of COPD patients according to their PAP and severity of disorder

**PAP (mm Hg)**	**Less than 40 mmHg**		**More than 40 mm Hg**		**Total**	

	**Smoker**	**Non-smoker**	**Smoker**	**Non-smoker**	**Smoker**	**Non- smoker**
**Disease severity**						
**Mild**	0	0	0	0	0	0
**Moderate**	3 (27.3%)	1 (11.1%)	4 (8.5%)	4 (9.3%)	7 (12.1%)	5 (9.6%)
**Severe**	4 (36.4%)	4 (44.4%)	14 (29.8%)	21 (48.8%)	18 (31%)	25 (48.1%)
**Very severe**	4 (36.4%)	4 (44.4%)	29 (61.7%)	18 (41.9%)	33 (56.9%)	22 (42.3%)
**Total**	11 (55%)	9(45%)	47(52.2%)	43(47.8%)	58(52.8%)	52(47.2%)

**Table 2.** Demographic and cardiopulmonary characters of COPD patients with pulmonary hypertension

	**Smoker**		**Non-smoker**	

	**Mean**	**S.D**	**Mean**	**S.D**
**Age**	67.5	9.4	70.8	8.1
**FEV1 (% predicted)**	27.8	12.9	31.8	12.4
**PAP (mmHg)**	53.9	13.3	52.6	15.1
**Hb (mg/dl)**	15.3	1.6	15.3	1.5
**PaO_2_ (mmHg)**	53.1	10.9	54.31	14.8

In patients with COPD and PH who had smoking history, there was a significant correlation between PAP and PaO_2_ (r= −0,291; P = 0.047), but there was no significant correlation between PAP and hemoglobin (r= −0.091; P= 0.541), PAP and FEV1 (r = −0.049; P= 0.746).

In patients with COPD and PH without past smoking history, there was a significant correlation between PAP and FEV1 (r = =0.341; P = 0.025), but there was no significant correlation between PAP and PaO_2_ (r= −0.271; P = 0.079), PAP and hemoglobin (r= −0.146; P = 0.351). Also, there was no significant correlation between hemoglobin and PAP, hemoglobin and FEV1, PaO_2_ and FEV1 in patients with and without smoking history (Tables 3–5).

**Table 3.** Correlation between PAP and Hb in COPD patients with pulmonary hypertension

	**Correlation coefficient**	**P-value**
**Smoker**	−0.091	0.541
**Non-smoker**	−0.146	0.351

**Table 4.** Correlation between Hb and FEV1 in COPD patients with pulmonary hypertension

	**Correlation coefficient**	**P-value**
**Smoker**	−0.156	0.294
**Non- smoker**	−0.121	0.439

**Table 5.** Correlation between PaO_2_ and FEV1 in COPD patients with pulmonary hypertension

	**Correlation coefficient**	**P-value**
**Smoker**	−0.045	0.765
**Non- smoker**	−0.072	0.645

## DISCUSSION

In patients with COPD who had smoking history there was a significant correlation between PAP and arterial oxygen pressure (PaO_2_). This result is in accordance with Chaouat et al. study (9), but mean of PAP in the present study was 53 mmHg, while in patients of Chaouat et al. study, it was less than 40 mmHg. This difference might be due to PAP measuring methods and also PH definition. In the present study echocardiography was used for measuring PAP, while in Chaouat et al. study, right heart catheterization was used (9).

Arcasoy et al. reported that there was minor difference in PAP when measured by echocardiography or right heart catheterization in patients with PAP less than 45 mmHg, but when PAP was higher than 45 mmHg, its value was more on echocardiography as compared to right heart catheterization (15). In the present study, PAP was measured by echocardiography. Majority of the patients were in very severe stages of the disease and mean FEV1 was 27.8% of predicted values. Lower FEV1 might be the other cause of higher measured PAP in these patients.

In patients with COPD and pulmonary hypertension without past smoking history, PAP had no significant correlation with PaO_2_, but PAP had a correlation with FEV1. Most of the patients with COPD and severe hypoxemia (PaO_2_ < 55 mmHg) have PH and in this situation mean PAP has a correlation with PaO_2_. PH is also seen in mild to moderate hypoxemia (PaO_2_ > 55mmHg), but in this group PAP has more correlation with FEV1 than PaO_2_ (16). In the present study, oxygenation of non-smoker patients with COPD was higher than smoker patients with COPD and this could be the explanation for these results. On the other hand, PH was seen at higher FEV1 in non-smoker patients in comparison with smoker patients with COPD. Sandoval et al. in their study on women with COPD due to biomass smoke inhalation reported that these patients rapidly progress to symptomatic disease and onset of PH was in patients with higher FEV1 (17). Same pattern was seen in patients of the present study. Moreira et al. reported that PH in smoker patients with COPD started earlier than other patients with COPD (18). In another study, there was no difference in the onset of PH in smoker and non-smoker patients with COPD (19).

Pulmonary parenchymal lesions were seen with airway lesions in COPD due to biomass smoke (20). In addition to emphysematous changes, interstitial involvement such as ground glass opacity, interlobular septum thickening and fibrotic bands were seen on High-Resolution Computed Tomography (HRCT) (21,22). Rivera et al. compared lung pathology of smoker patients with COPD and non-smoker patients with COPD due to biomass smoke. In the latter group, they observed fibrotic changes and anthracotic pigmentation in pulmonary tissue and vascular intima (23).

It seems that airway lesions combined with interstitial involvement can cause earlier PH in non-smoker patients with COPD and in this group vascular obliteration plays a more important role than hypoxia in the pathogenesis of PH.

In the present study there was no correlation between PAP and hemoglobin concentration. There are few studies on this topic. Nakamura et al. in their retrospective study on 41 emphysematous patients reported that hemoglobin level has independent effect on pressure and resistance of pulmonary artery (24). It is assumed that higher hemoglobin level can increase PAP due to hyper viscosity. But despite this hypothesis, frequency of PH was not higher in patients with polycythemia vera and high hemoglobin levels. It was shown that occurrence of PH in these patients is due to thromboembolic events (25). Thus, polycythemia alone has not great role in PH unless other accompanying risk factors are present. Correlation between PH and polycythemia should be clarified by more researches in the future.

There was no significant relationship between FEV1 and PaO_2_ or hemoglobin level in the present study. Although patients with COPD usually have hypoxia in severe stages, mild to moderate hypoxia may be seen in the earlier stages of the disease and there is no significant correlation between FEV1 and PaO_2_. Since the main mechanism for higher hemoglobin level in these patients was hypoxia and there was no significant relationship between FEV1 and PaO_2_, and relationship between FEV1 and hemoglobin was not expected.

## CONCLUSION

Several mechanisms have been considered for PH pathogenesis in patients with COPD, but exact mechanism is not clear. In smoker patients with COPD, hypoxemia plays main role but it seems that in non-smoker patients with COPD, degree of pulmonary parenchymal lesions and bronchial obliteration plays a more important role than hypoxia in the pathogenesis of pulmonary hypertension.
